# Intravenous Injections of Human Mesenchymal Stromal Cells Modulated the Redox State in a Rat Model of Radiation Myelopathy

**DOI:** 10.1155/2015/432369

**Published:** 2015-08-23

**Authors:** Jing Zhang, Lian-Bing Li, Zhu Qiu, Hong-Bo Ren, Jia-Yan Wu, Tao Wang, Zhong-Hui Bao, Ji-Fan Yang, Ke Zheng, Shao-Lin Li, Li Wei, Hua You

**Affiliations:** ^1^The First Affiliated Hospital of Liaoning Medical University, No. 2 Renmin Street, Guta District, Jinzhou, Liaoning 121000, China; ^2^Key Laboratory of Birth Defects and Reproductive Health of The National Health and Family Planning Commission, Chongqing Population and The Family Planning Science and Technology Research Institute, Chongqing 400020, China; ^3^Molecular Oncology and Epigenetics Laboratory, The First Affiliated Hospital of Chongqing Medical University, Chongqing 400016, China; ^4^Department of Oncology, The Second People's Hospital of Banan, Chongqing 400054, China; ^5^Department of Obstetrics and Gynecology, Xinqiao Hospital, The Third Military Medical University, Chongqing 400038, China; ^6^Department of Orthopedics II, Xi'An Gao Xin Hospital, Xi'an 710075, China; ^7^Department of Radiation Medicine, College of Basic Medical Sciences, Chongqing Medical University, Chongqing 400016, China; ^8^Affiliated Hospital of The Academy of Military Medical Sciences, Beijing 100071, China

## Abstract

The main aim of the present study was to assess the antioxidative effects of human umbilical cord-derived mesenchymal stromal cells (UC-MSCs) in a rat model of radiation myelopathy. UC-MSCs were isolated from Wharton's jelly (WJ) of umbilical cords. An irradiated cervical spinal cord rat model (C2-T2 segment) was generated using a ^60^Co irradiator to deliver 30 Gy of radiation. UC-MSCs were injected through the tail vein at 90 days, 97 days, 104 days, and 111 days after-irradiation. Histological damage was examined by cresyl violet/Nissl staining. The activities of two antioxidant enzymes catalase (CAT) and glutathione peroxidase (GPX) in the spinal cord were measured by the biomedical assay. In addition, the levels of vascular endothelial growth factor (VEGF) and angiopoietin-2 (Ang-2) in the spinal cord were determined by ELISA methods. Multiple injections of UC-MSCs through the tail vein ameliorated neuronal damage in the spinal cord, increased the activities of the antioxidant enzymes CAT and GPX, and increased the levels of VEGF and Ang-2 in the spinal cord. Our results suggest that multiple injections of UC-MSCs via the tail vein in the rat model of radiation myelopathy could significantly improve the antioxidative microenvironment in vivo.

## 1. Introduction

The spinal cord is a well-known example of “late-reacting” tissue in response to irradiation [1]. Exposure of the spinal cord to radiation can result in radiation myelopathy, which is a rare but serious complication of radiotherapy for cancer [2]. The late effects of radiation myelopathy (i.e., those occurring 6 months to several years after treatment) can be extremely severe and may seriously decrease the patient's quality of life. These effects are particularly dangerous because they are generally irreversible [3, 4]. Oxidative stress begins at radiation exposure and is sustained throughout the disease's progression, presumably through the radiation-induced activation of oxidant-generating enzymes, mitochondrial leakage, and the activation of the respiratory burst in the phagocytic cells that infiltrate damaged tissue [5, 6]. Therefore, antioxidant treatment strategies are being developed to treat the late effects of radiation myelopathy.

Recently, multipotent mesenchymal stromal cell (MSCs) treatment has attracted special attention for their antioxidative effects [7]. Upregulated reactive oxygen species (ROS) of carbon tetrachloride- (CCl_4_-) treated liver cells was attenuated by coculturing with MSCs. MSCs significantly increased superoxide dismutase (SOD) activity and inhibited ROS production in the injured liver. The gene expression levels of heme oxygenase-1 (Hmox-1), Bax inhibitor-1 (BI-1), hepatocyte growth factor (HGF), glutathione transferase (GST), and nuclear factor-erythroid 2 p45 subunit-related factor 20 (Nrf2), attenuated by CCl_4_, were increased up to basal levels after MSC transplantation.

The primary aim of the present study was to assess the antioxidative effects of human umbilical cord-derived mesenchymal stromal cells (UC-MSCs) in a rat model of radiation myelopathy. To the best of our knowledge, this is the first clinically based translational study to assess and highlight the antioxidative effects of human MSCs therapy for radiation myelopathy.

## 2. Materials and Methods

### 2.1. Animals

Adult female Sprague-Dawley (SD) rats (160–200 g, Laboratory Animal Center, Academy of Military Medical Sciences, Beijing, China) were housed and cared for according to the guidelines for the care and use of laboratory animals of the NIH and Academy of Military Medical Sciences (Beijing, China). All experimental procedures were approved by the Committee for Animal Use at the Academy of Military Medical Sciences. Every effort was made to minimize the number of animals used as well as their suffering. Water and food were available ad libitum in the cages.

### 2.2. Isolation and Differentiation of Human UC-MSCs

UC-MSCs were successfully isolated from Wharton's jelly (WJ) of umbilical cords according to the described methods in our previous report [8]. Briefly, fresh umbilical cords were collected after obtaining consent from the mothers. The umbilical cords were rinsed in phosphate-buffered saline (PBS) until the cord blood was cleared, and the blood vessels were removed. The remaining WJ tissue was cut into 1-2 mm^3^ pieces and placed in six-well plates in the presence of 0.1% collagenase type II (Sigma, USA) in PBS at 37°C for 1 h. Ten percent fetal bovine serum (FBS, Invitrogen, USA) was then added to stop the digestion. The dissociated mesenchymal cells were dispersed in 10% FBS-DMEM and further cultured until well-developed colonies of the fibroblast-like cells reached 80% confluence. Then, the cultures were trypsinized with 0.25% trypsin-EDTA (Invitrogen, USA) and passaged into new flasks for further expansion.

### 2.3. Irradiation and UC-MSCs Injection

According to our previous report [8], a total of 20 SD rats were randomly divided into 4 groups (*n* = 5 each): normal control (control) group, untreated irradiation (irradiation) group, PBS treatment (PBS) group, and UC-MSC treatment (UC-MSC) group. Each rat in the irradiation, PBS, and UC-MSC groups was anesthetized using 3% sodium pentobarbiturate (45 mg/kg) delivered by intraperitoneal injection and subsequently irradiated using a ^60^Co source. A ^60^Co irradiator (Model GWXJ80, NPIC, Chengdu, China) was used to conduct gamma ray irradiation, and the rats were irradiated with 30 Gy at a dose rate of approximately 150 cGy/min [8, 9]. The beam was strictly limited to a 2 cm segment of the cervical spine field spanning C2-T2. Rats in the UC-MSC group were injected with UC-MSCs (1 × 10^6^ cells for each injection and 4 × 10^6^ cells for 4 injections) injections through the tail vein in a 200 *μ*L volume at 90, 97, 104, and 111 days after irradiation. Rats in the PBS group were injected with PBS in a 200 *μ*L volume according to UC-MSC group treatment scheme. Rats in the irradiation group were not provided with any treatment after irradiation. Rats in the control group were fed normally and not irradiated during the same period. The rats were observed daily for up to 180 days after irradiation.

### 2.4. Specimen Processing and Histology

At 180 days after irradiation, the C2-T2 spinal cord segment was removed, and a 1 cm long central section of this segment was cut and immersed in 4% paraformaldehyde for histological analysis. The remaining portion of the spinal cord was frozen in dry ice power and stored at −80°C prior to use. The removed C2-T2 spinal cord segment was dehydrated in ethanol and embedded in paraffin. Some of the sections (5 *μ*m thickness) were deparaffinized, rehydrated, and stained with 0.1% cresyl violet at 37°C for 10 min. The stained sections were subsequently dehydrated, mounted, and observed under a light microscope. In the present work, we used frozen tissues from our previous study [8].

### 2.5. Biochemical Assays

Some frozen spinal cords were rinsed, weighed, and then homogenized in ice-cold saline for 10 min. The homogenates were collected after centrifugation at 15000 rpm for 15 min at 4°C, and the supernatants were used for the measurement of the total protein, catalase (CAT) activity, and glutathione peroxidase (GPX) activity via biomedical assay. The CAT activity and GPX activity in the spinal cord were measured using commercially available kits according to the manufacturer's protocols (Nanjing Jiancheng Bioengineering Institute, China). The CAT activity was determined based on the reaction of the enzyme with methanol in the presence of an optimal concentration of hydrogen peroxide. The GPX activity was determined using a spectrophotometric assay based on quantifying the rate of oxidation of glutathione (GSH) to glutathione disulfide (GSSG) by H_2_O_2_, catalyzed by GPX. The enzyme activity was expressed in nanomoles per milligram of protein.

### 2.6. ELISA

The levels of vascular endothelial growth factor (VEGF) and angiopoietin-2 (Ang-2) in the spinal cord tissues were determined using commercially available ELISA kits according to the manufacturer's protocols (VEGF: Abnova, Taipei city, Taiwan; Ang-2: EiAab, Wuhan, China). The OD value was determined by an ELISA reader at a wavelength of 450 nm and calculated in the linear part of the curve.

### 2.7. Statistics

All quantitative data are expressed as the mean value ± SEM. Differences were evaluated using one-way ANOVA. Comparisons between values at two time points were performed using the least significant difference (LSD) procedure. A *P* value <0.05 was considered statistically significant.

## 3. Results

### 3.1. UC-MSCs Ameliorated Irradiation-Induced Neuronal Damage in the Spinal Cord

**Figure 1:**
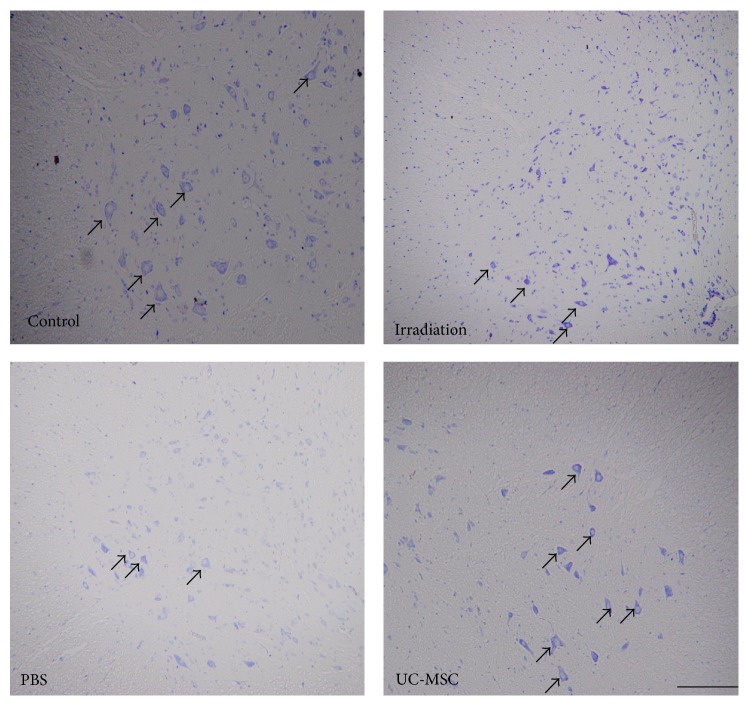
UC-MSC administration ameliorated irradiation-induced neuronal damage in the spinal cord. Representative micrographs of cresyl violet/Nissl-stained rat spinal cords at 180 days after irradiation following systemic injection of UC-MSCs. Arrows indicate Nissl bodies. Scale bars = 100 *μ*m.

We first identified that the neurons in the spinal cord were mainly injured using a single 30-Gy dose of irradiation [9]. We then observed that the UC-MSC-treated animals showed better pathological improvement in the neurons compared with PBS-treated and untreated irradiated animals in the present study. A markedly thickened cytoplasm, significant swelling and distension of the stroma, and hazy Nissl bodies in the neurons were not obvious in UC-MSC-treated animals (Figure 1).

### 3.2. UC-MSCs Increased the Activities of the Antioxidant Enzymes in the Spinal Cord

**Figure 2:**
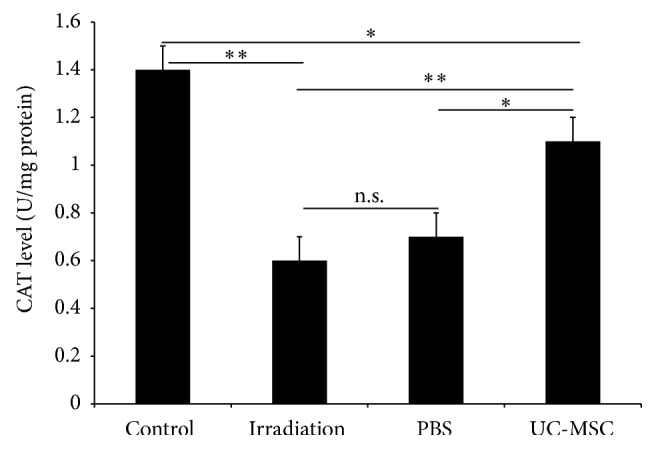
UC-MSC administration increased the activity of the antioxidant enzyme CAT in the spinal cord at 180 days after irradiation (*n* = 5). Error bars represent the S.E.M. Single asterisk represents *P* < 0.05 and double asterisk indicates *P* < 0.01. n.s., not significant.

**Figure 3:**
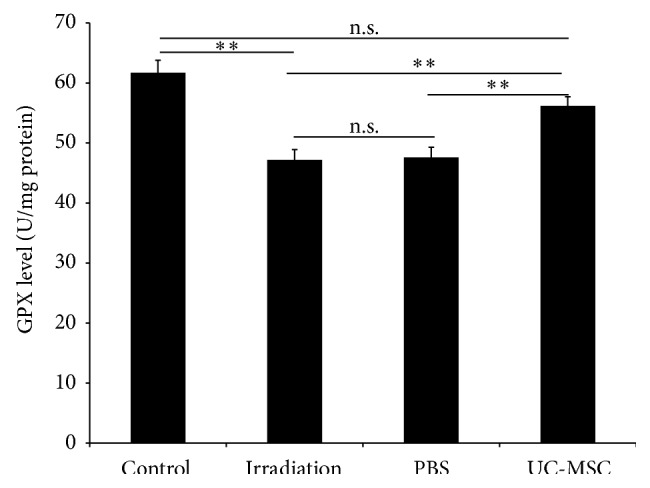
UC-MSC administration increased the activity of the antioxidant enzyme GPX in the spinal cord at 180 days after irradiation (*n* = 5). Error bars represent the S.E.M. Double asterisk indicates *P* < 0.01. n.s., not significant.

We examined whether the UC-MSCs treatment modulates the redox balance after irradiation in vivo. We measured the activities of two antioxidant enzymes, CAT and GPX, in the spinal cord using biochemical methods. The irradiated rats exhibited lower activities of CAT ((0.6 ± 0.1) U/mgprot) and GPX ((47.2 ± 1.7) U/mgprot) in the spinal cord compared with the normal controls (CAT: (1.4 ± 0.1) U/mgprot; GPX: (67.1 ± 2.1) U/mgprot) 180 days after irradiation. The activities of CAT ((1.1 ± 0.1) U/mgprot) and GPX ((56.2 ± 1.5) U/mgprot) in the spinal cord of the UC-MSC-treated animals were significantly higher than those of the PBS-treated animals (CAT: (0.7 ± 0.1) U/mgprot; GPX: (47.6 ± 1.7) U/mgprot) and the untreated irradiation animals (CAT: (0.6 ± 0.1) U/mgprot; GPX: (47.2 ± 1.7) U/mgprot) 180 days after irradiation. The activities of CAT and GPX in the spinal cord of the PBS-treated animals were similar to those of the untreated irradiation animals (Figures 2 and 3).

### 3.3. UC-MSCs Increased the Secretion of VEGF and Ang-2 in the Spinal Cord

**Figure 4:**
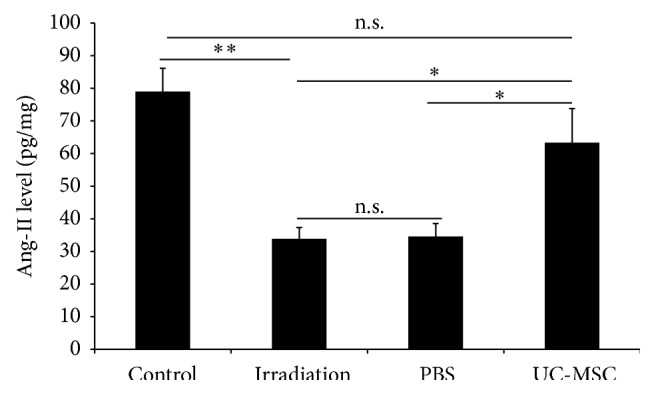
UC-MSC administration increased the level of Ang-2 in the spinal cord at 180 days after irradiation (*n* = 5). Error bars represent the S.E.M. Single asterisk represents *P* < 0.05 and double asterisk indicates *P* < 0.01. n.s., not significant.

**Figure 5:**
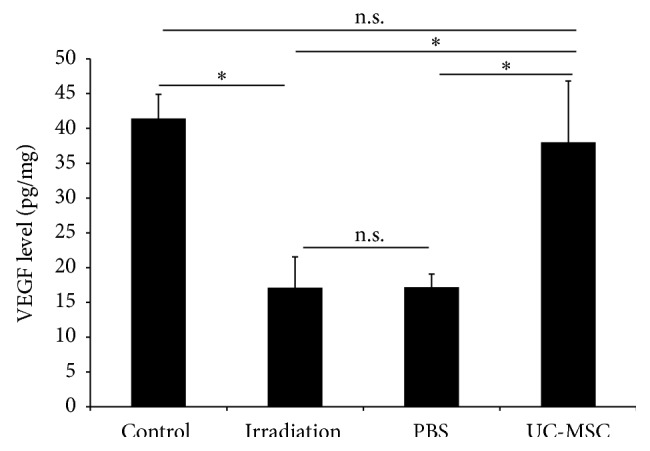
UC-MSC administration increased the level of VEGF in the spinal cord at 180 days after irradiation (*n* = 5). Error bars represent the S.E.M. Single asterisk represents *P* < 0.05. n.s., not significant.

We also examined whether the UC-MSCs treatment could increase the secretion of VEGF and Ang-2 after irradiation in vivo. We measured the levels of two potential promotion angiogenesis factors, VEGF and Ang-2, in the injured spinal cord using ELISA methods. The irradiated rats exhibited lower levels of VEGF ((17.1 ± 4.4) pg/mg) and Ang-2 ((33.9 ± 3.4) pg/mg) in the spinal cord compared with the normal controls (VEGF: (41.4 ± 3.5)  pg/mg; Ang-2 (79.0 ± 7.1) pg/mg) 180 days after irradiation. The levels of VEGF ((38.0 ± 8.8)  pg/mg) and Ang-2 ((63.4 ± 10.5) pg/mg) in the spinal cord of the UC-MSC-treated animals were significantly higher than those of the PBS-treated animals (VEGF: (17.2 ± 1.9) pg/mg; Ang-2 (34.6 ± 4.0) pg/mg) and the untreated irradiation animals (VEGF: (17.1 ± 4.4) pg/mg; Ang-2 (33.9 ± 3.4) pg/mg) 180 days after irradiation. The levels of VEGF and Ang-2 in the spinal cord of the PBS-treated animals were similar to those of the untreated irradiation animals (Figures 4 and 5).

## 4. Discussion

The primary aim of the present study was to assess the antioxidative effects of UC-MSCs in a rat model of radiation myelopathy. In the present study, we demonstrated that the administration of UC-MSCs through the tail vein ameliorates neuronal damage, increases the activities of the antioxidant enzymes CAT and GPX, and increases the levels of VEGF and Ang-2 in irradiation damage spinal cord. Our data not only defines the antioxidative roles of UC-MSCs infusion through the tail vein but also provides the further evidence that UC-MSCs have promotion angiogenesis effects in a rat model of radiation myelopathy.

MSCs have been demonstrated to increase the density of new blood vessels in traumatized spinal cord, resulting in functional recovery [10, 11]. As discussed by Oudega [12], this effect was induced by secreted factors exerting paracrine effects on local endothelial cells; MSCs might affect blood vessels directly by secreting growth factors such as BDNF, vascular endothelial growth factor (VEGF), and basic fibroblast growth factor (bFGF) or indirectly via factors that stimulate parenchymal cells to secrete blood vessel growth-regulating factors. We demonstrated that UC-MSC injection increases the endothelial cell density and the microvessel density in the white and gray matter of the spinal cord and increases spinal cord blood flow [8].

In the present study, the neurons of UC-MSC-treated irradiated animals were less damaged than those of untreated irradiated animals and PBS-treated animals. We also observed that UC-MSC injection significantly decreased the forelimb paralysis at 180 days after irradiation [8]. We interpreted this finding that the UC-MSC treatment fosters the preservation of the spinal cord parenchyma compared with PBS treatment or untreatment. The positive relationship between histological improvement and functional recovery had been well established.

MSCs themselves have antioxidative effects [7]. Our results confirmed the finding that UC-MSC increases the activities of two antioxidant enzymes, CAT and GPX, in the spinal cord. CAT is a common enzyme found in nearly all living organisms exposed to oxygen. It catalyzes the decomposition of hydrogen peroxide to water and oxygen. It is a very important enzyme in the biological defense system [13]. GPX converts hydrogen peroxide and lipid hydroperoxides into water, using GSH as an electron donor [14]. This increase in the activities of CAT and GPX in the spinal cord combated the deleterious effects of ROS.

In our study, we further provided evidence that UC-MSC injection increases the levels of two potential promotion angiogenesis factors, VEGF and Ang-2, in the irradiation injured spinal cord. VEGF is widely recognized to be a potential therapeutic target for regulating angiogenesis in the injured spinal cord [15]. VEGF has been shown to exert neuroprotective effects in damaged nervous tissue [16]. Interestingly, among the known stem cell-active chemokines, the angiogenic factor VEGF promotes mobilization and recruitment of endothelial and hematopoietic stem cells into the neoangiogenic sites, thereby accelerating the revascularization process. Vascular damage is considered the key step in the development of radiation myelopathy [8, 9]. Hence, the increasing level of VEGF in the irradiation injured spinal cord has been considered to be beneficial to the late effects of radiation myelopathy.

It has been also shown that Ang-2 has a proangiogenic role in the injured CNS [17], and higher Ang-2 level contributes to the better functional recovery after spinal cord injury [18]. Ang-2 is stored in intracellular Weibel-Palade bodies in endothelial cells along with von Willebrand factor, which is involved in haemostasis [19]. Stored Ang-2 has a half-life of 18 h but can be secreted within minutes of stimulation [19]. In adults, Ang-2 secretion is induced by hypoxia and various cytokines such as VEGF and basic fibroblast growth factor, hypoxic factor HIF1*α*, inflammatory mediators tumor necrosis factor *α* and NF*κ*B, or vasoactive molecule thrombin [20]. Increasing level of VEGF and hypoxia microenvironment in irradiation spinal cord [21] activates the Ang-2 secretion and finally contributes to better functional recovery.

It is well known that hypoxia microenvironment is intimately related to oxidative stress. In fact, although cell oxygen availability is reduced, ROS is overproduced in hypoxia due to a change of mitochondrial ROS production rate causing an increase of leakage of electrons from the mitochondrial respiratory chain. On the other hand, cells are able to specifically respond to hypoxia by activating several transcription factors, in particular, HIF1*α*. HIF1*α* in turn activates the expression of adaptive genes, such as VEGF, which is crucial for angiogenesis and for a vital recovery to an optimal oxygen supply [22].

## 5. Conclusions

In summary, we have demonstrated that multiple injections of UC-MSCs via the tail vein in the rat model of radiation myelopathy could significantly improve the antioxidative microenvironment in vivo. To the best of our knowledge, this is the first clinically based translational study to assess and highlight the antioxidative effects of human MSCs therapy for radiation myelopathy.
